# Specificity Evaluation and Disease Monitoring in Arthritis Imaging with Complement Receptor of the Ig superfamily targeting Nanobodies

**DOI:** 10.1038/srep35966

**Published:** 2016-10-25

**Authors:** Fang Zheng, Harris Perlman, Patrick Matthys, Yurong Wen, Tony Lahoutte, Serge Muyldermans, Shemin Lu, Patrick De Baetselier, Steve Schoonooghe, Nick Devoogdt, Geert Raes

**Affiliations:** 1 Department of Biochemistry and Molecular Biology, Health Science Center, Xi’an Jiaotong University, Xi’an 710061, P. R. China; 2 Research group of Cellular and Molecular Immunology, Vrije Universiteit Brussel, Brussels, B-1050, Belgium; 3 Laboratory of Myeloid Cell Immunology, VIB Inflammation Research Center, Ghent, B-9052, Belgium; 4 Division of Rheumatology, Feinberg School of Medicine, Northwestern University, Chicago, IL60611, USA; 5 Laboratory of Immunobiology, Rega Institute, Katholieke Universiteit Leuven, Leuven, B-3000, Belgium; 6 Center for Translational Medicine, School of Life Science and Technology, Xi’an Jiaotong University, Xi’an 710049, P. R. China; 7 In Vivo Cellular and Molecular Imaging Center, Vrije Universiteit Brussel, Brussels, B-1090, Belgium; 8 Department of Nuclear Medicine, UZ Brussel, Vrije Universtiteit Brussel, Brussels, B-1090, Belgium

## Abstract

Single-photon emission computed tomography combined with micro-CT (SPECT/μCT) imaging using Nanobodies against complement receptor of the Ig superfamily (CRIg), found on tissue macrophages such as synovial macrophages, has promising potential to visualize joint inflammation in experimental arthritis. Here, we further addressed the specificity and assessed the potential for arthritis monitoring. Signals obtained with ^99m^Tc-labelled NbV4m119 Nanobody were compared in joints of wild type (WT) versus CRIg^−/−^ mice with collagen-induced arthritis (CIA) or K/BxN serum transfer-induced arthritis (STIA). In addition, SPECT/μCT imaging was used to investigate arthritis development in STIA and in CIA under dexamethasone treatment. ^99m^Tc-NbV4m119 accumulated in inflamed joints of WT, but not CRIg^−/−^ mice with CIA and STIA. Development and spontaneous recovery of symptoms in STIA was reflected in initially increased and subsequently reduced joint accumulation of ^99m^Tc-NbV4m119. Dexamethasone treatment of CIA mice reduced ^99m^Tc-NbV4m119 accumulation as compared to saline control in most joints except knees. SPECT/μCT imaging with ^99m^Tc-NbV4m119 allows specific assessment of inflammation in different arthritis models and provides complementary information to clinical scoring for quantitatively and non-invasively monitoring the pathological process and the efficacy of arthritis treatment.

Rheumatoid arthritis (RA) is a chronic destructive inflammatory disease of the joints, featuring a mixed mononuclear and granulocytic cellular infiltration in the inflamed joints^1^. Over the past years, RA treatment has shifted from controlling symptoms with non-steroidal anti-inflammatory drugs and glucocorticoids such as dexamethasone, to controlling the disease process with anti-proliferative or cytotoxic drugs like methotrexate, alone or in combination with biologics^2^,^3^.

Rodent arthritis models have contributed greatly to the overall knowledge of RA and allow preclinical assessment of novel diagnostic and therapeutic interventions^3^. Type II collagen induced arthritis in DBA/1 mice (CIA) represents the current gold standard and has been used extensively for evaluation of novel therapies^3^. CIA requires specific disease-susceptible inbred mouse strains (DBA/1 and B10.RIII) and places a heavy emphasis on the early inductive phase of disease^4^. However, most transgenic and knockout strains of mice are in a C57BL/6J background (H-2^b^), which is regarded to be relatively resistant to arthritis induction^5^. In this context, it has been reported that C57BL/6J mice are indeed susceptible to arthritis induced by chicken type II collagen with a modified immunization protocol. The development of collagen induced arthritis is associated with a very strong T cell response that remains elevated for up to 3 months post-immunization^6^. KRN mice expressing the KRN transgenic T cell receptor and MHC class II molecule Ag7 develop severe inflammatory arthritis^7^. Transfer of arthritogenic serum of KRN and nonobese diabetic (K/BxN) mice leads to induction of robust and reproducible disease in several mouse strains (K/BxN serum-transfer-induced arthritis [STIA])^8^. Interestingly, the sterile inflammatory arthritis of STIA is transient, often spontaneously resolving after 15–30 days. Similar to human arthritis, STIA features high expression of pathogenic autoantibodies to glucose-6-phosphate isomerase (GPI), making this model ideal for the identification of the effector pathways involved in the arthritis process^9^.

Standard non-invasive readouts of disease severity such as paw thickness/volume or clinical scores mainly emphasize the oedema component of arthritic disease^10^. Other non-invasive readouts to monitor disease evolution or treatment efficacy, such as near-infrared, PET or SPECT imaging and scintigraphy, possibly combined with anatomical imaging techniques such as X-ray, ultrasonography and magnetic resonance imaging, can provide information on underlying molecular and cellular inflammatory processes as well as bone destruction. Tracers used for molecular arthritis imaging include ^18^F-fluoro-2-deoxy-D-glucose for visualizing glucose metabolism and ^99m^Tc-methylenediphosphonate for monitoring bone turnover^11^. Various stroma-targeting tracers have been described for imaging arthritic synovitis, including ^99m^Tc-anti-E-selectin-Fab for targeting activated endothelium^12^, radiolabelled antibodies targeting fibroblast activation protein for detection of fibrobolast-like synoviocytes^13^ or probes targeting activated macrophages such as ^18^F-polyethyleneglycol-folate^14^,^15^ or ^111^In-anti-F4/80-A3-1^16^. In addition, radiolabeled biologicals such as anti-CD3, anti-CD4, anti-CD20 and anti-TNF have been evaluated for imaging RA^13^. As new tools being introduced in this field, Nanobodies are tracers combining the specificity of antibodies with the pharmacokinetics of small molecules, thus yielding high contract images within hours after inoculation^17^. In a preclinical setting, Nanobodies allow convenient SPECT imaging after standard tricarbonyl-chemistry-based labelling with 99mTc on their carboxy-terminal hexahistidine-tag^18^, distant from the antigen-binding site and thus not interfering with antigen recognition^19^.

In an approach of targeting markers expressed on macrophages for non-invasive inflammation tracking, we reported before that radiolabelled Nanobodies targeting the macrophage mannose receptor (NbMMR), initially described as effective probes for *in vivo* imaging of tumor-associated macrophages in hypoxic tumor areas^20^,^21^, also accumulate in joints of CIA mice^22^. Similarly, joint accumulation of the anti-CRIg Nanobody NbV4m119, as determined via SPECT/μCT imaging and quantification of radioactivity in dissected joints, correlates with arthritic scores and reflects disease severity over a range of scores in different joints^23^. Whereas osteoclasts and macrophages express similar levels of MMR^22^, CRIg is readily detected on macrophages but not osteoclasts (data not shown). Immunofluorescence microscopy has revealed that NbV4m119 specifically targets a subset of CD68-positive macrophages in synovium of DBA/1 CIA mice^23^. In line with a lower signal in non-arthritic joints, and thus a higher contrast for NbV4m119 as compared to NbMMR, accumulation of ^99m^Tc-NbV4m119 in knees of mice developing CIA could even be detected before the onset of macroscopic clinical symptoms.

In concordance with CRIg expression on tissue macrophages such as Kupffer cells, ^99m^Tc-NbV4m119 uptake in the liver was shown to be higher as compared to a non-targeting control Nanobody in WT but not CRIg^−/−^ C57BL/6J mice^23^. In the current study, we further confirmed the specificity of CRIg targeting by NbV4m119 in arthritic joints of WT versus CRIg^−/−^ C57BL/6J CIA mice. In addition, STIA was induced through transfer of arthritogenic serum of K/BxN mice, progeny of KRN mice, transgenic for a T cell receptor recognizing an epitope of bovine RNase, and nonobese diabetic mice^7^,^8^. Interestingly, the sterile inflammatory arthritis of STIA is transient, spontaneously resolving after 15–30 days^9^, thus allowing assessment of ^99m^Tc-NbV4m119-based monitoring of the onset, development and recovery of arthritis symptoms of C57BL/6J STIA mice. Finally, we also used ^99m^Tc-NbV4m119 to monitor dexamethasone treatment effects in DBA/1 CIA mice.

## Results

### Immunofluorescence microscopy to assess macrophage-specific NbV4m119 targeting in joints of collagen-induced arthritis mice

As a first step in addressing the target specificity at the level of the cell-type expressing CRIg that is being targeted by the NbV4m119, CIA was induced in DBA/1 mice and immunofluorescence microscopy was performed on tissue sections of decalcified ankles exhibiting different arthritic scores. Especially in sections of ankles exhibiting higher arthritic scores, staining by the NbV4m119 could be detected (see Supplementary Fig. S0). Moreover, the staining pattern of NbV4m119 overlapped with that one of an antibody targeting the macrophage marker F4/80, indicating that NbV4m119 is indeed binding to macrophages in the arthritic joints.

### Specificity of NbV4m119 targeting CRIg in C57BL/6J mice with CIA

**Figure 1:**
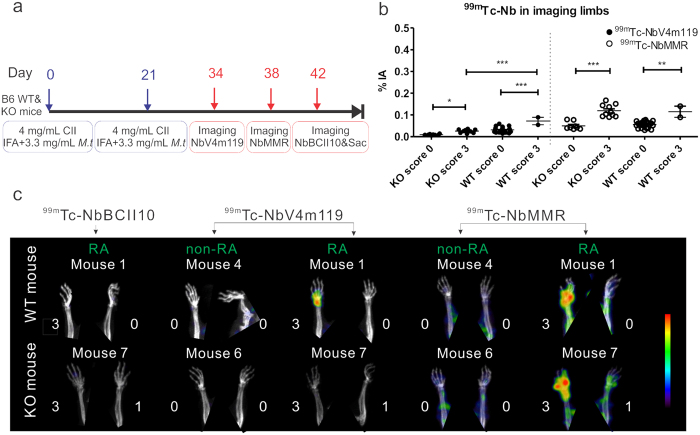
SPECT/CT quantification and imaging of ^99m^Tc-NbV4m119, ^99m^Tc-NbMMR or ^99m^Tc-NbBCII10 accumulation in WT and CRIg^−/−^ (KO) C57BL/6J mice with CIA after the onset of the arthritis symptoms. (**a**) Experimental timelines for C57BL/6J mice with CIA; IFA = Incomplete Freund’s Adjuvant; M.t = Mycobacterium tuberculosis strain H37RA; Sac = mice were sacrificed. SPECT/micro-CT imaging was performed 3 h post intravenous injection of ^99m^Tc-labeled NbV4m119, NbMMR or NbBCII10. (**b**) Signals obtained in the joints with score 3 and joints with score 0 were grouped in four symptomatic and four asymptomatic mice (two WT and two KO mice in each group). Data are expressed as mean values of % of injected activity (%IA) of ^99m^Tc-Nanobodies obtained from the limbs (front paws, metatarsal joints and ankles). **p < 0.01 and ***p < 0.001. (**c**) Representative SPECT/μCT image of front paws in CIA mice, 3 h post injection with ^99m^Tc-labeled NbV4m119, NbMMR or NbBCII10. Clinical scores are indicated next to each joint. Representative images of 1 mouse per group are shown using National Institutes of Health color scale and are scaled to maximum in the whole image. The data are representative of two independent experiments.

Another approach to assess specificity of targeting, is to compare targeting in WT versus CRIg^−/−^ mice. Since CRIg^−/−^ mice are not available in the DBA/1 background and C57BL/6J mice are relatively resistant to CIA^5^, we used a modified immunization protocol to induce CIA in WT and CRIg^−/−^ C57BL/6J mice (Fig. 1a). Yet even when using this protocol, both WT and CRIg^−/−^ C57BL/6J mice showed a low disease incidence (2 out of 7 mice developing symptoms in each group) as evaluated in two separate experiments and in fact, arthritic scores of individual paws tended to be even lower in CRIg^−/−^ C57BL/6J as compared to WT C57BL/6J mice (see Supplementary Fig. S1a). As depicted in the experimental time lines in Fig. 1a, imaging using ^99m^Tc-NbV4m119 was performed at day 34 in the four symptomatic mice and four asymptomatic mice (two WT and two CRIg^−/−^ C57BL/6J mice). As had been documented and quantified before^23^, ^99m^Tc-NbV4m119 targeted CRIg specifically in liver of naïve and arthritic WT mice, but not in CRIg^−/−^ mice (see whole body images in Supplementary Fig. S1b). Signals that were detected for ^99m^Tc-NbV4m119 in the kidneys and the bladder were also observed for a control, non-targeting ^99m^Tc-NbBCII10 and are not attributable to specific targeting, but to the fast filtration of unbound Nanobody from the bloodstream through the renal route. As far as the uptake in joints is concerned, in WT mice ^99m^Tc-NbV4m119 showed higher uptake in inflamed joints as compared to asymptomatic joints (Fig. 1b, left of the dotted line). In contrast, inflamed joints of CRIg^−/−^ C57BL/6J mice (score 3 as compared to score 0) only showed a slightly higher uptake of ^99m^Tc-NbV4m119 that may be due to joint oedema (Fig. 1b, left of the dotted line). Moreover, when comparing joints with the same arthritic score 3, ^99m^Tc-NbV4m119 signals were much higher in WT versus CRIg^−/−^ mice, indicating the antigen-specificity of these signals.

A reduced accumulation of NbV4m119 in CRIg^−/−^ mice as compared to WT mice could not only be due to the absence of CRIg as a target in these mice, but could also be related to a functional effect of the absence of CRIg on the intensity of the arthritic inflammation or oedema that would affect the tissue penetration of Nanobodies in general, independent of the target specificity. To exclude this possibility we also included NbMMR in this study as a Nanobody of which the target is still expected to be present in CRIg^−/−^ mice. Thereby, the uptake of ^99m^Tc-NbMMR was quantified via SPECT/μCT imaging in the same mice at day 38 post immunization (see experimental time lines in Fig. 1a). As reported before^22^, NbMMR targets MMR in several organs and tissues, including myocardium, bone marrow, spleen and liver, but also accumulates in joints of CIA mice (see Supplementary Fig. S1b). Notably, and in contrast to the results obtained with ^99m^Tc-NbV4m119, uptake of ^99m^Tc-NbMMR was enhanced in inflamed versus asymptomatic joints in both WT mice and CRIg^−/−^ C57BL/6J mice (Fig. 1b, right of the dotted line), arguing against a reduced joint inflammation in CRIg^−/−^ CIA mice being the cause for the lower ^99m^Tc-NbV4m119 uptake. Furthermore, reconstructed SPECT/μCT images revealed hot spots of radioactive signals only in symptomatic wrists in WT mice injected with ^99m^Tc-NbV4m119 and WT or CRIg^−/−^ mice injected with ^99m^Tc-NbMMR, but not in ^99m^Tc-NbV4m119 injected CRIg^−/−^ mice or in mice injected with a control, non-targeting NbBCII10 (at day 42 post immunization), confirming specificity of the ^99m^Tc-NbV4m119 signals (see cropped images focusing on front paws in Fig. 1c). Similar conclusions were obtained for signals in other joints such as ankles or knees (see whole body images in Supplementary Fig. S1c).

### Specificity of NbV4m119 targeting in STIA C57BL/6J mice

**Figure 2:**
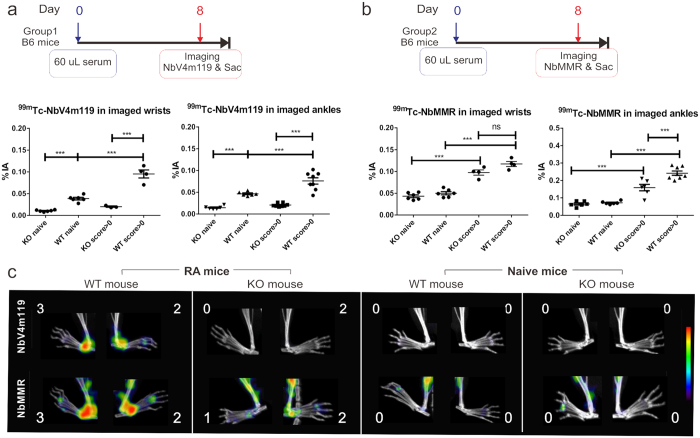
*In vivo* SPECT/CT imaging with an anti-CRIg tracer (NbV4m119) or an anti-MMR tracer in arthritic joints of C57BL/6J WT and CRIg^−/−^ (KO) mice with STIA. Experimental timelines and quantification of tracer accumulation in wrist and ankle joints of C57BL/6J mice with STIA which were injected with KBxN serum and monitored via SPECT/micro-CT imaging using ^99m^Tc-NbV4m119 (**a**) or ^99m^Tc-NbMMR (**b**) at day 8 post serum transfer. For each tracer, 4 KO and WT mice were injected with serum and 3 naïve KO and WT mice were used as control. Imaging & Sac = mice were imaged and sacrificed. The % of injected activity (%IA) was quantified for the wrist or ankle area in SPECT imaging (mean ± SEM, ***p < 0.001, ns: not significant). Naive: naive mice without serum injection; Score > 0: the ankles with arthritis score 1, 2 or 3. (**c**) Representative SPECT/μCT image of STIA C57BL/6J mice, 3 h post injection with ^99m^Tc-labeled NbV4m119 or ^99m^Tc-labeled NbMMR. Clinical scores are indicated next to each joint. Representative images of ankles in 1 out of 4 mice per group are shown using National Institutes of Health color scale and are scaled to maximum in the whole image.

Next, STIA was induced in C57BL/6J mice, whereby 8 days later one group of mice was used for imaging using ^99m^Tc-NbV4m119 and another group of mice was used for imaging using ^99m^Tc-NbMMR (see experimental time lines in Fig. 2a). Symptoms were mainly observed in the ankles, whereas some of the wrists seemed to remain asymptomatic at day 8 (see Supplementary Fig. S2a,S2b). For imaging with ^99m^Tc-NbV4m119, 4 WT and 4 CRIg^−/−^ serum injected mice at day 8 (all mice developing symptoms) were compared to 3 WT and 3 KO naive mice. The mean levels of ^99m^Tc-NbV4m119 uptake in wrists and ankles of WT arthritic mice, which developed symptoms, were higher as compared to naive WT and CRIg^−/−^ mice (Fig. 2a). The signal of ^99m^Tc-NbV4m119 uptake in symptomatic wrists and ankles of CRIg^−/−^ mice was not different as compared to non-symptomatic joints or naive joints in CRIg^−/−^ mice. The same conclusions were obtained via measurement of radioactivity in dissected joints of euthanized mice as compared to quantification of the imaging data (see Supplementary Fig. S2c). Representative images cropped to focus on hind legs are shown in Fig. 2c, and whole body images including also front paws are shown in Supplementary Fig. S2e, highlighting focal signals of ^99m^Tc-NbV4m119 uptake in ankles of arthritic WT mice, but not of naïve WT or arthritic or naïve CRIg^−/−^ mice (Fig. 2c).

A different set of mice was imaged with the ^99m^Tc-NbMMR, whereby 4 WT and 4 CRIg^−/−^ serum injected mice at day 8 were compared to 3 WT and 3 CRIg^−/−^ naive mice. The wrists and ankles showed significantly higher signals in symptomatic WT mice (score > 0) as compared to naive WT mice (Fig. 2b,c and Supplementary Fig. S2d). Of note, in this experiment the symptomatic CRIg^−/−^ mice had a lower score in ankles than the WT mice, suggesting CRIg deficiency may (variably) affect arthritis development in these mice (Supplementary Fig. S2b). Accordingly, ^99m^Tc-NbMMR uptake was significantly higher in the ankle joints but not in wrists of symptomatic WT as compared to symptomatic CRIg^−/−^ mice. Yet, in contrast to the results obtained with NbV4m119, NbMMR uptake in CRIg^−/−^ mice was increased in ankles and wrists of symptomatic mice as compared to naive mice (Fig. 2b,c and Supplementary Fig. S2d,S2f), indicating that the lack of accumulation of ^99m^Tc-NbV4m119 in ankles and wrists of symptomatic versus asymptomatic CRIg^−/−^ mice truly reflects the absence of specific targeting to inflamed joints in the KO mice rather than merely reduced inflammation in joints of CRIg^−/−^ mice.

### Using ^99m^Tc-NbV4m119 for monitoring arthritis progression in STIA

**Figure 3:**
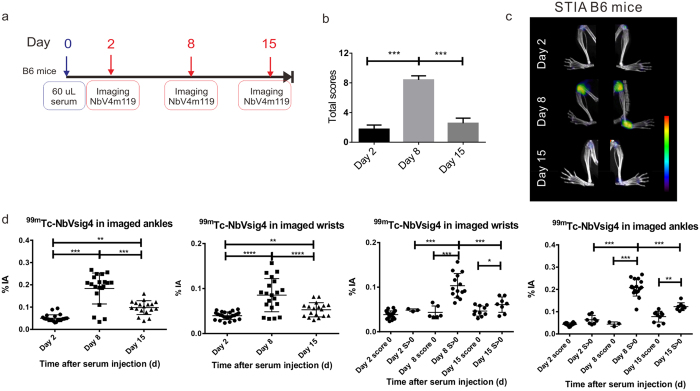
*In vivo* SPECT/CT imaging with an anti-CRIg tracer for monitoring arthritis recovery in STIA C57BL/6J mice. (**a**) Experimental timelines for 12 WT mice injected with KBxN serum and imaged at day 2, day 8 and day 15 post serum transfer. Imaging = SPECT/μCT imaging. (**b**) The clinical arthritic scores (additive scores for all 4 paws of each mouse, expressed as group mean ± SEM) were measured at day 2, day 8 and day 15 after immunization. (**c**) Representative SPECT/μCT image of ankles in STIA serum transfer arthritis mice, 3 h post injection with ^99m^Tc-labeled NbV4m119. The same arthritic mice displaying symptoms of arthritis showed specific uptake of ^99m^Tc-labeled NbV4m119 in inflamed joints on day 2, day 8 and day 15 post serum injection. Clinical scores are indicated next to each joint. Representative images of 1 out of 12 mice per group are shown using National Institutes of Health color scale and are scaled to maximum in whole image. (**d**) Uptake of ^99m^Tc-NbV4m119 in individual wrists and ankles of mice. The % of injected activity (%IA) was quantified for the wrist area. score 0: the wrists with score 0; S > 0: the wrists with arthritis score 1, 2 or 3. The % of injected activity (%IA) was quantified for the ankle area. (mean ± SEM, *P < 0.05, **P < 0.01, *** P< 0.001, ns: not significant).

Next, we evaluated ^99m^Tc-NbV4m119 for monitoring the initial induction and later recovery of RA symptoms in STIA. 12 C57BL/6J mice were injected with K/BxN serum on day 0. Mice were imaged with ^99m^Tc-NbV4m119 at 2 days (onset of RA), 8 days (peak of RA) and 15 days (recovering phase of RA) after initial immunization (Fig. 3a). The scores of these mice increased from day 2 to day 8 and subsequently decreased after 15 days (Fig. 3b and Supplementary Fig. S3a). Significant ^99m^Tc-NbV4m119 uptake was mainly observed in inflamed joints in hind legs (Fig. 3c) and in front paws (see Supplementary Fig. S3b) of arthritic mice. When assessing probe accumulation in individual wrists and ankles, ^99m^Tc-NbV4m119 uptake at day 8 was significantly increased as compared to day 2 (Fig. 3d). In line with the observation that the symptoms were reduced in mice beyond day 8, the ^99m^Tc-NbV4m119 accumulation in the wrists and ankles significantly decreased at day 15 as compared to day 8 (Fig. 3d). These results demonstrate that ^99m^Tc-NbV4m119 can sense the CRIg level *in vivo* from the onset, peak and recovery of arthritis.

### Monitoring the effect of dexamethasone therapy for RA using ^99m^Tc-NbV4m119 in CIA DBA/1 mice

**Figure 4:**
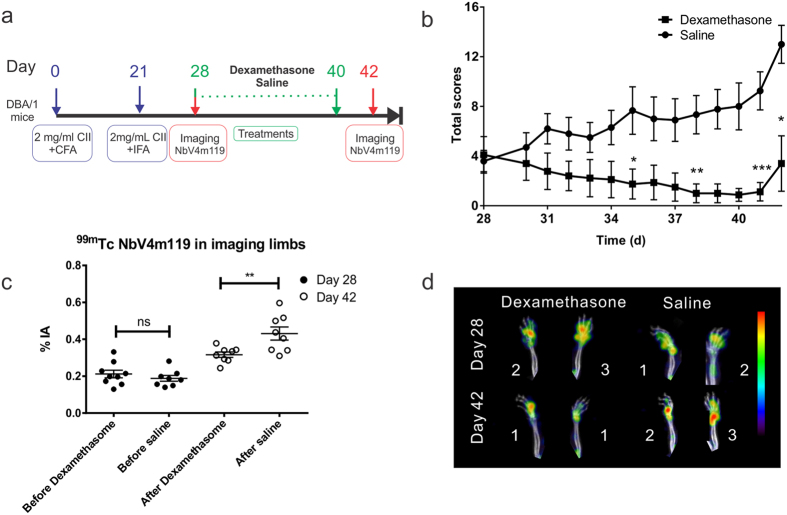
Monitoring the clinical scores and uptake of ^99m^Tc-NbV4m119 in the four limbs of DBA/1 mice with CIA following dexamethasone therapy. (**a**) Experimental timelines for the CIA therapy monitoring experiment; CFA = Complete Freund’s Adjuvant; IFA = Incomplete Freund’s Adjuvant; Imaging = SPECT/μCT imaging; Sac = mice were sacrificed. Mice (n = 10) were immunized with type II collagen in complete Freund’s adjuvant. The treatments were started at 28 days and finished at 41 days after initial immunization. Treatments consisted of daily i.p. injection with 100 μl saline control or 0.25 mg/kg dexamethasone. (**b**) The clinical additive scores of the RA mice were measured until 42 days after immunization. *p < 0.05, **p < 0.01, and ***p < 0.001. (**c**) SPECT/CT image quantification of ^99m^Tc-NbV4m119 in all 4 limbs of CIA DBA/1 mice (sum of ankles and front paws) at 28 days and 42 days after immunization were quantified as %IA. Data are expressed as mean ± SEM. ns: not significant, **p < 0.01. (**d**) Representative SPECT/μCT image of front paws in CIA DBA/1 mice, 3 h post injection with ^99m^Tc-labeled NbV4m119. The same arthritic mice displaying symptoms of arthritis showed specific uptake of ^99m^Tc-labeled NbV4m119 in front paws on 28 days and 42 days post serum injection. Clinical scores are indicated next to each joint. Representative images of front paws in 1 out of 10 per group CIA DBA/1 mice are shown using National Institutes of Health color scale and are scaled to maximum in whole image.

Finally, we evaluated the use of ^99m^Tc-NbV4m119 for monitoring the effects of therapeutic intervention. In order to obtain a maximum sensitivity for the effect of the therapeutic treatment, we used the gold-standard model of CIA in arthritis-sensitive DBA/1 mice for these experiments. Two groups of mice (n = 10) were compared to allow monitoring the effect of the dexamethasone treatment: one group received dexamethasone i.p. daily 28 days after arthritis-induction and the second group was injected with saline as control. Each group was imaged with ^99m^Tc-NbV4m119 at day 28 (before treatment) and day 42 (after treatment) after initial immunization (Fig. 4a). Before the start of treatment at day 28, mice in both groups had an average cumulative arthritic score of 4. In contrast to the saline injected group, the scores of the mice in the dexamethasone treated group decreased to nearly baseline treatment till day 40. We also noticed that as soon as the dexamethasone treatment stopped on day 41, the arthritis scores of this group rebounded again on day 42 (Fig. 4b). The total ^99m^Tc-NbV4m119 accumulation in four limbs of mice in the dexamethasone treated group (at 42 days post immunization) was significantly lower than in the saline treated group, which correlated with decreasing clinical scores (Fig. 4c,d). Similar conclusions were obtained in ankles, but no significant difference was detected among knees of dexamethasone treated and control mice. Hot spots of ^99m^Tc-NbV4m119 accumulation were observed in knees of both groups of mice (see Supplementary Fig. S4). Notably, in all joints, the recorded imaging readout values in the dexamethasone injected group at day 42 remained at least as high as those recorded at day 28 (i.e. before the onset of therapy) (Fig. 4c).

## Discussion

Molecular imaging of arthritis, which includes anatomical imaging techniques such as ultrasonography and MRI or molecular imaging such as optical imaging or nuclear imaging, will improve the understanding of the pathogenesis and development of the disease. MRI imaging produces anatomically detailed images of cartilage, tendons, synovial membrane and bone which ultrasonography could not penetrate^11^. It provides a higher contrast resolution, allowing detection of synovial hyperplasia, cartilage and bone degradation, inflammation and osteitis and is able to show signs of early arthritis in patients^24^. Molecular imaging can provide molecular information on the underlying biochemical and inflammatory processes, that is complementary to anatomical imaging. Development of metal plasmonic nanoparticles for MRI^25^ allows using MRI also for molecular imaging in addition to its use for anatomical imaging. Other molecular probes being developed for molecular arthritis imaging include fluorescence and near-infrared labelled probes^26^ for optical imaging or radioisotopes labelled probes for scintigraphy, PET or SPECT^14^. Nuclear imaging techniques generate deeper tissue penetration and lower background than optical imaging techniques and are more sensitive than structural imaging^11^. In the current study, we built on previous results obtained with NbV4m119 as a probe for molecular imaging of arthritic inflammation^23^. Despite the low incidence of arthritis in the CIA model in C57BL/6J mice, we obtained antigen-specific ^99m^Tc-NbV4m119 signals in arthritic WT mice but not in arthritic CRIg^−/−^ mice. Also in the STIA model, which is readily inducible in C57BL/6J mice, ^99m^Tc-NbV4m119 specifically detected the inflamed lesions in the arthritic WT mice but not in the CRIg^−/−^ mice. Throughout these different experiments in the CIA and STIA models, hot spots of ^99m^Tc-NbV4m119 accumulation in arthritic joints of CRIg^−/−^ mice were never detected. Moreover, sustained joint accumulation of ^99m^Tc-NbMMR in CRIg^−/−^ mice indicates that the impaired accumulation of ^99m^Tc-NbV4m119 in symptomatic CRIg^−/−^ mice truly reflects the absence of specific targeting in CRIg^−/−^ mice, rather than reduced joint inflammation.

^99m^Tc-NbV4m119 signals provide a quantitative read-out for disease development and resolution in the STIA model. The detection of hot spots of NbV4m119 signal in some of the score 0 joints at day 2, suggests that the *in vivo* imaging is more sensitive than the classic scoring system. These findings offer perspectives for monitoring disease evolution, either spontaneous or under therapy, provided that baselines can be established allowing comparison of the obtained signals. Increased resolution when switching from SPECT to PET imaging and increased joint size in humans as compared to rodents, thereby hold potential for increased contrast upon clinical translation of such inflammation imaging.

Non-invasive imaging can monitor the same animal at different time points before or after the onset of arthritis, allowing to follow up a therapy or treatment. MRI-based outcome measures have recently been adopted by pharma companies for clinical trial research to measure clinical trial outcome of therapy such as anti-TNF agents, including adalimumab, infliximab, etanercept and golimumab. These enable earlier assessment of drug efficacy than radiographic outcomes^27^. In a recent study using ^111^In-labelled antibodies targeting fibroblast activation protein, macrophages and integrin αvβ3, decreased joint uptake of tracers was observed in arthritic mice undergoing etanercept treatment^28^. In the current study using ^99m^Tc-NbV4m119 in CIA DBA/1 mice receiving dexamethasone as therapy, the recorded signals at day 42 were lower in the dexamethasone injected group as compared to the saline control group, but remained at least as high as those recorded at day 28 (i.e. before the onset of therapy). At the level of the knees, wherein ^99m^Tc-NbV4m119-based imaging is a sensitive indicator of inflammation^29^, similar signals were even detected in the dexamethasone treated group as in the control group. These data are in line with the observation that the clinical scores rose immediately as soon as dexamethasone treatment stopped, confirming that dexamethasone is better at providing symptom relief than at altering disease progression^30^ and indicating that SPECT/μCT imaging can indeed provide complementary molecular information on the effect of the therapy on the underlying disease pathology in the various joints as compared to the clinical score.

Our studies have confirmed that Nanobodies targeting CRIg constitute a specific tool for non-invasive SPECT/CT imaging as a way of assessing inflammation in arthritis models *in vivo*. Moreover, the monitoring studies indicate that imaging arthritic mice using ^99m^Tc-NbV4m119 appears to serve as a non-invasive means for tracing disease evolution and response to therapy, providing complementary and more sensitive information as compared to paw swelling and clinical scoring.

## Materials and Methods

### Ethical approval

Animal studies were performed according to the European Community Council Directive 2010/63/EU and were approved by our university’s Ethical Committee for Animal Experiments (Approval N°12-220-6; 24/09/2012).

### Mice

Male C57BL/6J (8–12 week-old) and DBA/1 mice (8 week-old) were purchased from Janvier Sas. CRIg^−/−^ mice (C57BL/6J background) were generously provided by Genentech (San Francisco, CA).

### Arthritis models

CIA was induced in WT and CRIg^−/−^ C57BL/6J male mice using a modified immunization involving two 21-day-separated subcutaneous injections of 100 μL of 4 mg/mL chicken collagen type II (Sigma-Aldrich) in acetic acid, emulsified in incomplete Freund’s adjuvant with added 3.3 mg/mL heat-killed Mycobacterium tuberculosis strain H37RA (Difco Laboratories), at two sites (the base of the tail and a slightly more anterior location) after anesthesia. Each limb was scored for severity of arthritis for CIA as follows: 0 = normal; 1 = redness/swelling of one joint; 2 = redness/swelling of more than one joint; 3 = swelling of entire paw; 4 = ankylosis and/or deformity. For dexamethasone treatment efficacy monitoring and immunofluorescence microscopy, cohorts of DBA/1 mice were used for CIA induction as described^23^ and were treated daily with i.p. 0.25 mg/kg dexamethasone in 100 μL saline or saline control from day 28 to day 41.

STIA mice were sensitized via an i.v. injection in the tail of C57BL/6J mice with 60 μL of arthritogenic serum from K/BxN mice as described^8^. All paws were assigned a score for inflammation in STIA on a scale of 0 to 3: 1 = mild swelling of the ankle insufficient to reverse the normal V shape of the foot; 2 = swelling sufficient to make the ankle and midfoot approximately equal in thickness to the forefoot; 3 = reversal of the normal V shape of the foot. The thickness of the ankles and the wrists were monitored using a calliper across the ankle or wrist joint at the widest point.

### Nanobodies

Nanobodies against CRIg (NbV4m119), the β-lactamase BCII enzyme of *Bacillus cereus* (NbBCII10) and MMR (NbMMR) were generated as described^19^,^22^,^23^. For ^99m^Tc labelling, the Nanobodies were subcloned into the pHEN6c vector introducing a hexahistidine-tag. All Nanobodies were produced in, and purified from, the periplasm of *Escherichia coli* WK6 cells.

### ^99m^Tc-Nanobody labelling and pinhole SPECT/μCT analysis

Nanobodies were labelled with ^99m^Tc via the hexahistidine tag, purified via NAP-5 column (Sephadex G-25, GE Healthcare) and 0,22 μm membrane filtered as described^18^. Radiochemical purity was determined using instant thin-layer chromatography (Pall Corp., Life Sciences) in acetone solvent, revealing the amount of Nb-complexed and unincorporated ^99m^Tc at the application and the solvent front, respectively^18^. 52.8 ± 0.01 MBq of ^99m^Tc-Nbs activity in 80–100 μL (at 192 MBq/nmol) was injected. 3 h post injection, anesthetized mice were imaged using μCT (Skyscan 1178; Skyscan) followed by pinhole SPECT (e.cam180; Siemens Medical Solutions) as described^23^. Images were reconstructed as described^31^, using an iterative reconstruction algorithm and corrected for attenuation and scatter. Micro-CT images were reconstructed using filtered backprojection (NRecon; SkyScan). Images were viewed and analyzed using AMIDE: A Medical Image Data Examiner software^32^. A calibration factor, obtained by SPECT image analyses of syringes with known activities, is used to convert SPECT image pixels to radioactivity levels. Regions of interest were drawn, recorded amounts of radioactivity in these regions were divided by the amount of injected activity (corrected for decay) and expressed as percentage of injected activity (% IA).

To determine biodistribution *ex vivo*, mice were sacrificed and tissues were dissected and weighed, and their radioactivity content was measured using an automated γ-counter (Cobra II Inspector 5003; Canberra-Packard, Schwadorf, Austria). Organ uptake was calculated as the percentage of injected activity per gram (% IA/g) and corrected for decay.

### SPECT/μCT imaging time lines for the different arthritis models

(1) *In vivo* imaging monitoring the modified CIA model in WT (n = 2) and CRIg^−/−^ (n = 2) C57BL/6J mice. The imaging was performed at day 34, day 38 and day 42, using ^99m^Tc-NbV4m119, ^99m^Tc-NbMMR, or ^99m^Tc-NbBCII10 respectively. The clinical scores of the wrists and ankles were monitored. (2) *In vivo* imaging monitoring STIA at day 8. WT and CRIg^−/−^ C57BL/6J mice (n = 4, 14 weeks old) received the KBxN mouse serum administration. The clinical scores and the thickness of the wrists and ankles were monitored at day 8. The arthritic WT and CRIg^−/−^ mice were injected with ^99m^Tc-NbV4m119 or ^99m^Tc-NbMMR and imaged by SPECT/CT on day 8 post serum injection. After the anesthetized mice were imaged using μCT followed by pinhole SPECT, mice were sacrificed, wrists and ankles were dissected and weighed, and their radioactivity content was measured using an automated γ-counter. (3) *In vivo* imaging monitoring the STIA pathological process. WT C57BL/6J mice (n = 12, 10 weeks old) received the KBxN mice serum administration. The clinical scores and the thickness of the wrists and ankles were monitored till day 15. The arthritic WT C57BL/6J mice were injected with ^99m^Tc-NbV4m119 each time and imaged by SPECT/CT on day 2 (onset of RA), day 8 (peak of RA) and day 15 (RA partly resolved) post serum injection. Anesthetized mice were imaged using μCT followed by pinhole SPECT. (4) Treatment efficacy monitoring by ^99m^Tc-NbV4m119. For evaluation of the dexamethasone therapy by imaging, beginning on 28 days after the initial immunization, cohorts of CIA DBA-1 mice (n = 10) were treated i.p. with dexamethasone (daily) or received saline (daily) and served as control. The treatments were finished at day 41. Till day 42, the scores were monitored for each mouse every two days. The arthritic mice were injected with ^99m^Tc-NbV4m119 and imaged by SPECT/CT before (day 28) and after (day 42) the dexamethasone or saline treatments.

### Immunofluorescence microscopy

CIA was induced in DBA/1 mice. Mice were sacrificed under anesthesia and ankles exhibiting different arthritic scores were removed and fixed in 4% paraformaldehyde solution for 24 hours. Ankles were decalcified in 10% EDTA pH 7.4 (Sigma, USA) for 4 weeks (EDTA solution refreshed once a week) then embedded in paraffin. 8 μm sections were prepared from paraffin-embedded tissues. The sections were incubated in 1% blocking reagent (Roche) in PBS (1% PBS/BR) containing detection antibodies. Anti-F4/80(Cl:A3-1)/Alexa fluor 488 was purchased from AbD Serotec and anti-HA-tag (16B12)/Alexa Fluor 594 was purchased from Invitrogen. Each section was incubated overnight with 2 μg of antibody and Nb. Specific labelling was detected with anti-HA/Alexa fluor 549 in 1% PBS-BR. Fluoro-Gel II/DAPI (Electron Microscopy Sciences) mounted slides were used for fluorescence microscopy with a UPlanFI 10x, 20x or 30x objective on a OLYMPUS BX51 microscope with OLYMPUS DP71 CCD and OLYMPUS DP Controller software.

### Statistical analysis

Significance analysis of data was conducted using a two-tailed unpaired Student’s T test when two groups were analyzed or one-way ANOVA analysis of variance multiple–comparison post-test. Prism 6.0 (Graphpad software) was used for statistical analyses and graph creation. P-values ≤ 0.05 were considered significant.

## Additional Information

**How to cite this article**: Zheng, F. *et al*. Specificity Evaluation and Disease Monitoring in Arthritis Imaging with Complement Receptor of the Ig superfamily targeting Nanobodies. *Sci. Rep.* **6**, 35966; doi: 10.1038/srep35966 (2016).

## Supplementary Material

Supplementary Information
