# Supplementary material for: Specificity Evaluation and Disease Monitoring in Arthritis Imaging with Complement Receptor of the Ig superfamily targeting Nanobodies
Source: Sci Rep. 2016 Oct 25;6:35966. doi: 10.1038/srep35966 (PMC5078791; doi:10.1038/srep35966)
Supplement: Supplementary Information [file srep35966-s1.pdf]

Supplementary information for Zheng et al. (2016). "Specificity Evaluation and Disease Monitoring in Arthritis Imaging with Complement Receptor of the Ig superfamily targeting Nanobodies".

Fang Zheng[1,2,3], Harris Perlman[4], Patrick Matthys[5], Yurong Wen[6], Tony Lahoutte[7, 8], Serge Muyldermans[2], Shemin Lu[1], Patrick De Baetselier[2,3], Steve Schoonooghe[2,3]*, Nick Devoogdt[2,6]* and Geert Raes[2,3]*

[1]Department of Biochemistry and Molecular Biology, Health Science Center, Xi'an Jiaotong University, Xi'an 710061, P.R. China; [2]Research group of Cellular and Molecular Immunology, Vrije Universiteit Brussel, Brussels, B-1050, Belgium; [3]Laboratory of Myeloid Cell Immunology, VIB Inflammation Research Center, Ghent, B-9052, Belgium; [4]Division of Rheumatology, Feinberg School of Medicine, Northwestern University, Chicago, IL60611, USA; [5]Laboratory of Immunobiology, Rega Institute, Katholieke Universiteit Leuven, Leuven, B-3000, Belgium; [6]Center for Translational Medicine, School of Life Science and Technology, Xi'an Jiaotong University, Xi'an 710049, P.R. China; [7]In Vivo Cellular and Molecular Imaging Center, Vrije Universiteit Brussel, Brussels, B-1090, Belgium; [8]Department of Nuclear Medicine, UZ Brussel, Vrije Universtiteit Brussel, Brussels, B-1090, Belgium.

* S.S, N.D. and G.R. share senior authorship of this manuscript.
Correspondence and requests for materials should be addressed to G.R. (E-mail: Geert.Raes@vib-vub.be)

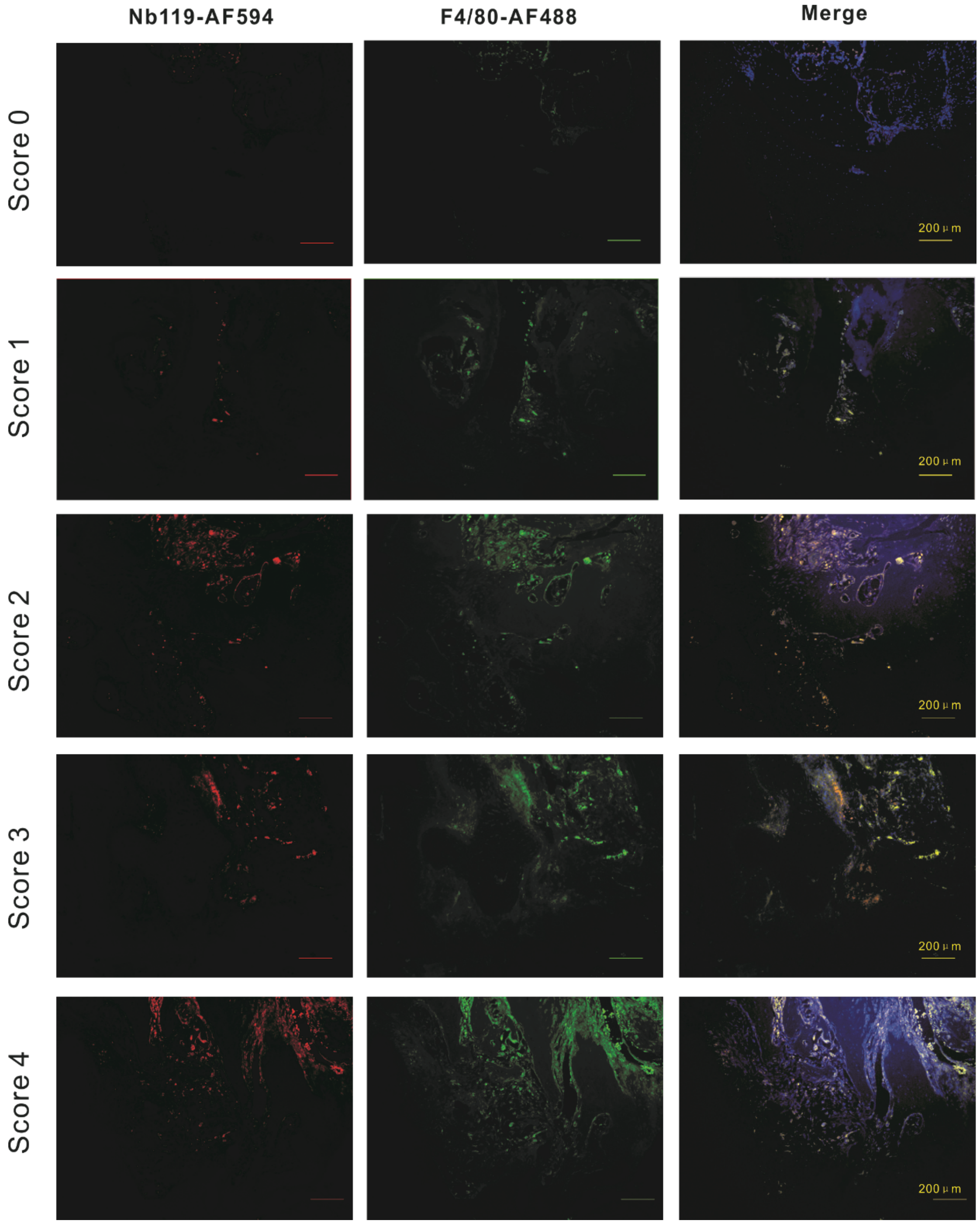

Supplementary Figure 0: *Immunofluorescence microscopy on joints of left hind paws of CIA mice having different clinical scores.* DBA-1 mice were immunized with type II collagen in complete Freund's adjuvant. The joints of left hind paws having different clinical scores were fixed in 4 % paraformaldehyde solution and decalcified in 10 % EDTA pH 7.4 for 4 weeks. Paraffin-embedded tissues were stained with Hemaglutinin (HA) -tagged Nb119 and Alexafluor 594-conjugated anti-HA antibody (left panels) and Alexafluor488-conjugated anti-F4/80 antibody (middle panels). The merged image (right panels) shows the co-localization of CRIg expression with F4/80^{+} macrophages. DAPI (blue) was used as a nuclear stain. The images were observed with a ×100 magnification and were obtained from 1 mouse that was representative of 9 tested in 3 individual experiments.

(a)

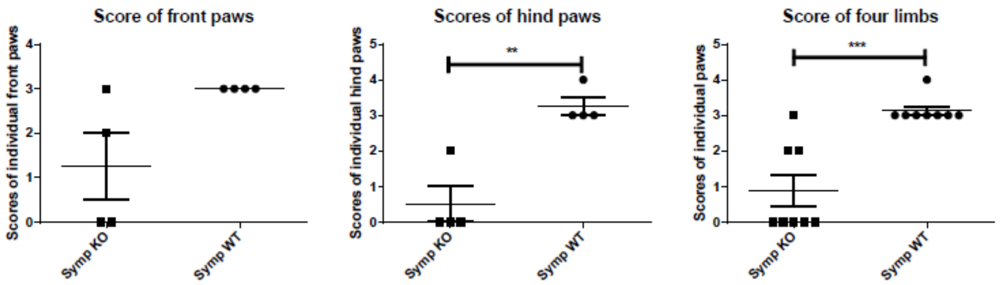

(b)

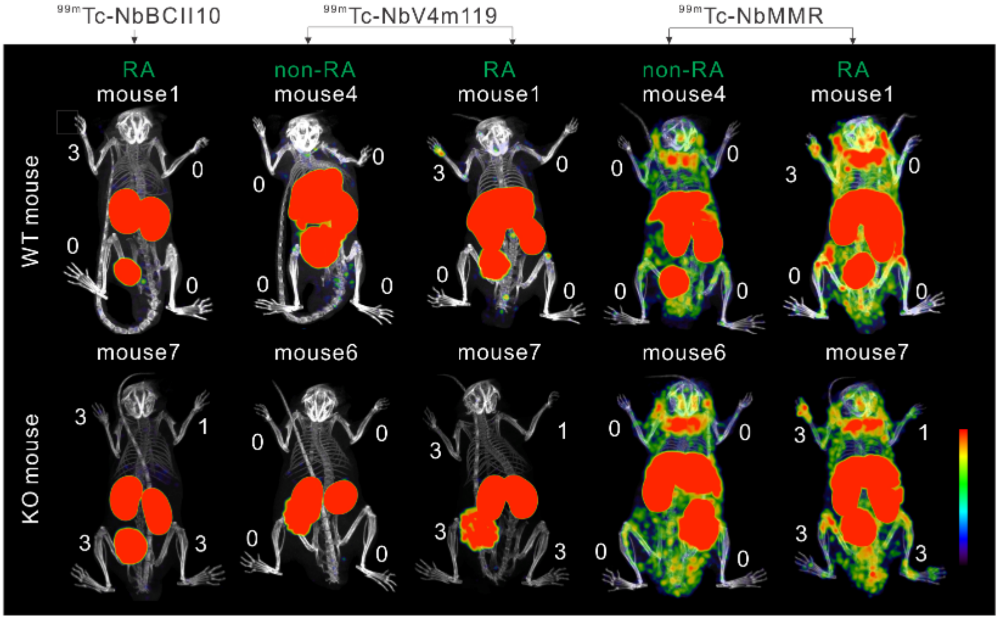

(c)

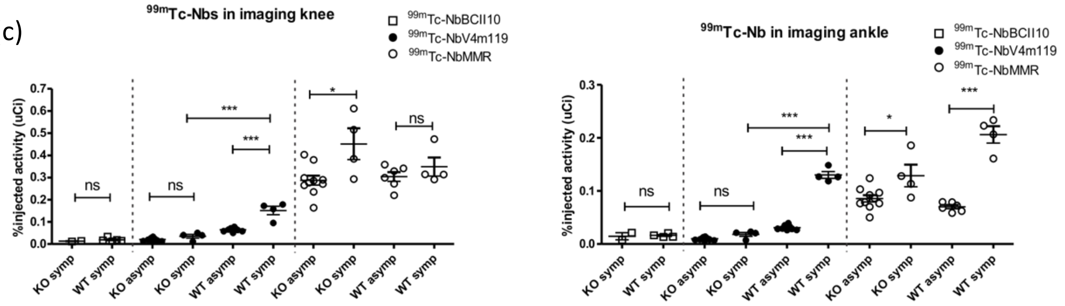

Supplementary Figure 1: **(a)** *Monitoring the scores of wrist or ankle joints of C57BL/6J mice.* CRIg$^{-/-}$ (KO) and wild type (WT) C57BL/6J mice were induced for CIA and the thickness and clinical scores were monitored at day 34 post initial injection. The scores of wrists (left), ankles (middle) and four limbs (right) of WT and KO mice are shown. n=4, mean± SEM are shown (**p<0.01, and ***p<0.001). **(b)** *Whole body SPECT/CT imaging of ^{99m}Tc-NbV4m119 or ^{99m}Tc-NbMMR accumulation in WT and CRIg$^{-/-}$ C57bl/6 mice after the onset of the arthritis symptoms*. Representative SPECT/μCT images of modified CIA mice, 3h post injection with ^{99m}Tc-labeled BCII10，NbV4m119 or NbMMR. Clinical scores are indicated next to each joint. Representative images of 1 out of 6 mice per group are shown using National Institutes of Health color scale and are scaled to maximum in the whole image. **(c)** *SPECT/CT quantification of ^{99m}Tc-NbV4m119, ^{99m}Tc-NbMMR or ^{99m}Tc-NbBCII10 accumulation in knees and ankles of WT and CRIg$^{-/-}$ (KO) B6 mice with CIA.* Signals obtained in the asymptomatic joints (asymp) and symptomatic joints (symp) were grouped in four symptomatic and four asymptomatic mice (two WT and two KO mice in each group). Data are expressed as mean values of % of injected activity (%IA) of ^{99m}Tc-Nanobodies obtained from joints or ankles. **: p<0.01 and ***: p<0.001.

Mice	Limbs	Arthritic status of limb	NbV4m119	NbMMR
WT mouse 1	Front paw R	Symptomatic	0.0890	0.0890
	Front paw L	Asymptomatic	0.0227	0.0645
	Hind paw R	Asymptomatic	0.0270	0.0662
	Hind paw L	Asymptomatic	0.0310	0.0736
WT mouse 2	Front paw R	Asymptomatic	0.0502	0.0611
	Front paw L	Symptomatic	0.0648	0.1403
	Hind paw R	Asymptomatic	0.0458	0.0616
	Hind paw L	Asymptomatic	0.0350	0.0610
WT mouse 3	Front paw R	Asymptomatic	0.0509	0.0301
	Front paw L	Asymptomatic	0.0594	0.0316
	Hind paw R	Asymptomatic	0.0367	0.0385
	Hind paw L	Asymptomatic	0.0401	0.0388
WT mouse 4	Front paw R	Asymptomatic	0.0432	0.0718
	Front paw L	Asymptomatic	0.0319	0.0509
	Hind paw R	Asymptomatic	0.0254	0.0740
	Hind paw L	Asymptomatic	0.0329	0.0699
KO mouse 1	Front paw R	Asymptomatic	0.0080	0.0311
	Front paw L	Asymptomatic	0.0083	0.0370
	Hind paw R	Asymptomatic	0.0070	0.0456
	Hind paw L	Asymptomatic	0.0118	0.0322
KO mouse 2	Front paw R	Asymptomatic	0.0080	0.0421
	Front paw L	Asymptomatic	0.0083	0.0397
	Hind paw R	Asymptomatic	0.0070	0.0483
	Hind paw L	Asymptomatic	0.0118	0.0422
KO mouse 3	Front paw R	Symptomatic	0.0330	0.1368
	Front paw L	Asymptomatic	0.0092	0.0775
	Hind paw R	Symptomatic	0.0173	0.0928
	Hind paw L	Symptomatic	0.0270	0.1135
KO mouse 4	Front paw R	Symptomatic	0.0297	0.1671
	Front paw L	Asymptomatic	0.0139	0.0789
	Hind paw R	Symptomatic	0.0247	0.0938
	Hind paw L	Symptomatic	0.0132	0.0999

Supplementary Table 1: *SPECT/CT imaging quantification of ^{99m}Tc-NbV4m119 or ^{99m}Tc-NbMMR accumulation in limbs of WT and CRIg$^{-/-}$ (KO) C57bl/6 mice after the onset of CIA.* Signals obtained in the asymptomatic joints (left aligned) and symptomatic joints (right aligned) were grouped from four symptomatic and four asymptomatic mice (two WT and two KO mice in each group). Data are expressed as mean values of % of injected activity (%IA) of ^{99m}Tc-Nanobodies obtained from right (R) or left (L) hind or front paws.

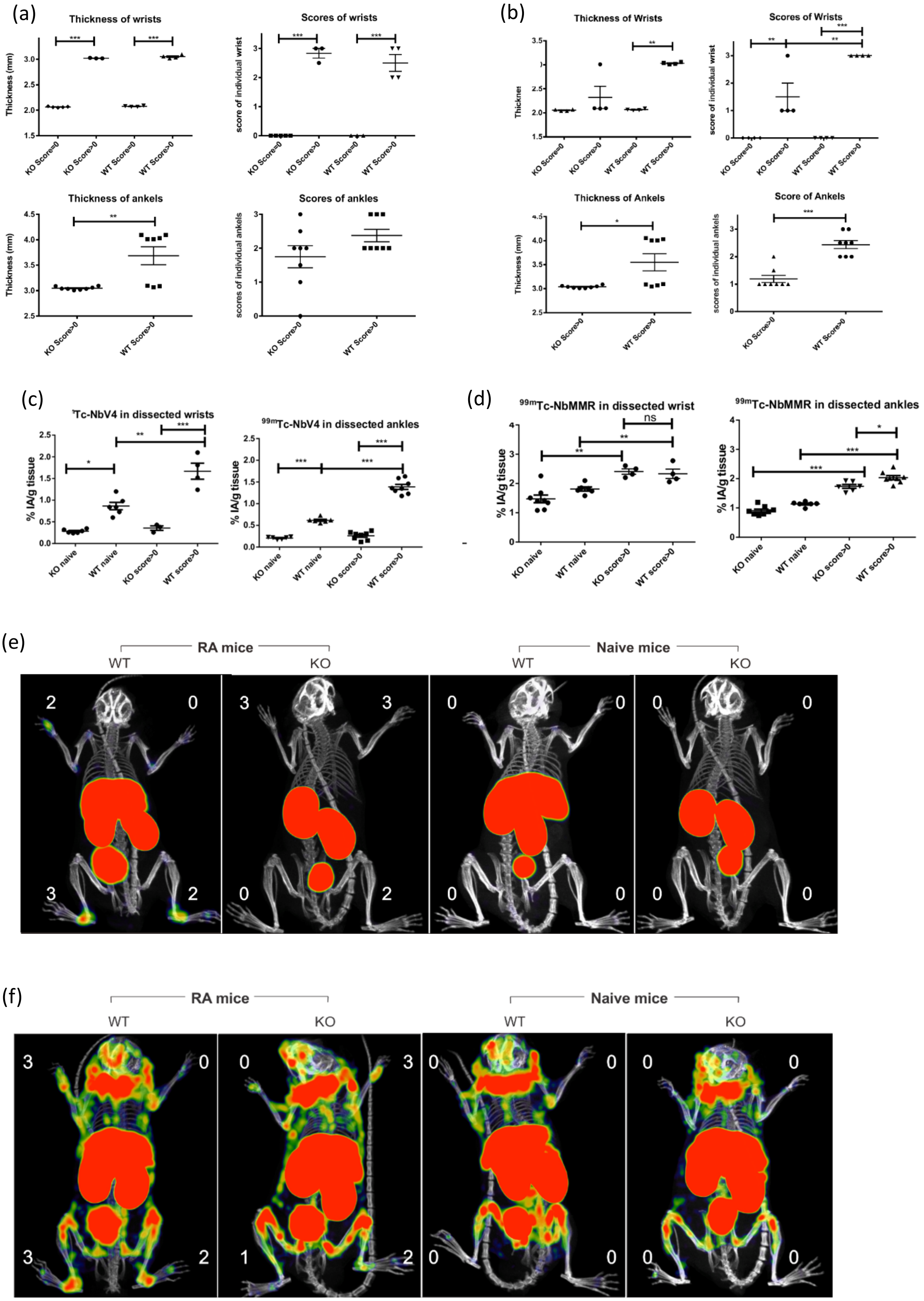

Supplementary Figure 2: **(a)** *Monitoring the thickness and the scores of wrist or ankle joints of KBxN serum transfer arthritis mice for the ^{99m}Tc-NbVsig4 imaging group.* 4 CRIg$^{-/-}$ (KO) and 4 wild type (WT) mice were injected with KBxN serum and the thickness and clinical scores were monitored at day 8 post serum injection. The thickness and the scores of wrists (**top row**) and the thickness and the scores of ankles (**bottom row**) of WT and KO mice were measured at day 8. n=4, mean± SEM are shown (**p<0.01, and ***p<0.001). **(b)** *Monitoring the thickness and the scores of wrist or ankle joints of KBxN serum transfer arthritis mice for the ^{99m}Tc-NbMMR imaging group.* 4 CRIg$^{-/-}$ (KO) and wild type (WT) mice were injected with KBxN serum and the thickness and clinical

scores were monitored at day 8 post serum injection. The thickness and the scores of wrists (**top row**) and the thickness and the scores of ankles (**bottom row**) of WT and KO mice were measured at day 8. n=4, mean± SEM are shown (, *p<0.05, **p<0.01, and ***p<0.001). **(c+d)** *Ex vivo radioactivity measurements in dissected ankles and wrists and in vivo SPECT/CT images of front paws with an anti-CRIg tracer (NbV4m119) or an anti-MMR tracer in arthritic joints of B6 WT and CRIg$^{-/-}$ (KO) mice with STIA.* Radioactive content of dissected wrists and ankles as measured in a γ-counter and expressed as percentage of injected activity per gram tissue (%IA/g) using ^{99m}Tc-NbV4m119 ***(c)*** or ^{99m}Tc-NbMMR ***(d)*** at day 8 post serum transfer. For each tracer, 4 KO and WT mice were injected with serum and 3 naïve KO and WT mice were used as control. (mean ± SEM, *: p< 0.05,** p< 0.01,***: p< 0.001, ns: not significant). Naive: naive mice without serum injection; Score>0: the ankles with arthritis score 1, 2 or 3. (C) Representative SPECT/µCT image of STIA B6 mice, 3h post injection with ^{99m}Tc-labeled NbV4m119 or ^{99m}Tc-labeled NbMMR. Clinical scores are indicated next to each joint. Representative images of front paws in 1 out of 4 mice per group are shown using National Institutes of Health color scale and are scaled to maximum in the whole image. **(e+f)** *In vivo SPECT/CT imaging with the anti-CRIg tracer NbV4m11 or the anti-MMR tracer NbMMR in arthritic joints of K/BxN serum transfer arthritis wild type (WT) mice and CRIg$^{-/-}$ (KO) mice*. Representative SPECT/µCT image of K/BxN serum transfer arthritis mice are shown, 3h post injection with ^{99m}Tc-labeled NbV4m119 ***(e)*** or ^{99m}Tc-labeled NbMMR ***(f)***. Clinical scores are indicated next to each joint. Representative images of 1 out of 4 mice per group are shown using National Institutes of Health color scale and are scaled to maximum in the whole image.

(a)

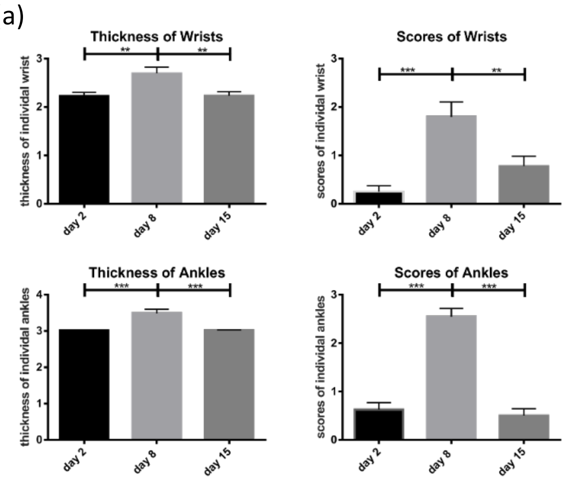

(b)

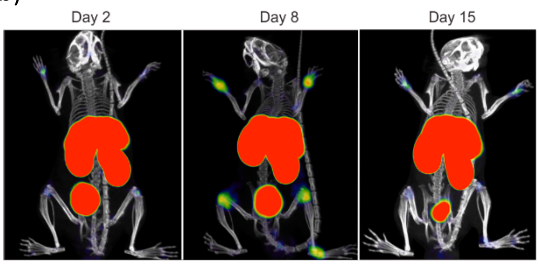

Supplementary Figure 3: **(a)** *Monitoring the thickness and the scores of wrist and ankle joints of KBxN serum transfer arthritis mice at day 0, day 8 and day 15 post serum injection***.** 12 WT B6 mice were injected with KBxN mice serum. The thickness and scores of wrists (**top**) and ankles **(bottom)** were measured in WT at day 2, day 8 and day 15 post serum injection. n=12, mean± SEM are shown (**p<0.01, and ***p<0.001). **(b)** *In vivo SPECT/CT imaging with an anti-CRIg tracer monitoring the recovering KBxN serum transfer arthritis mice.* Representative SPECT/µCT image of KBxN serum transfer arthritis mice, 3h post injection with ^{99m}Tc-labeled NbV4m119. The same arthritic mice displaying symptoms of arthritis showed specific uptake of ^{99m}Tc-labeled Vsig4 in inflamed joints on 2 days, 8 days and 15 days post serum injection. Representative images of 1 out of 12 mice per group are shown using National Institutes of Health color scale and are scaled to maximum in whole image.

(a)

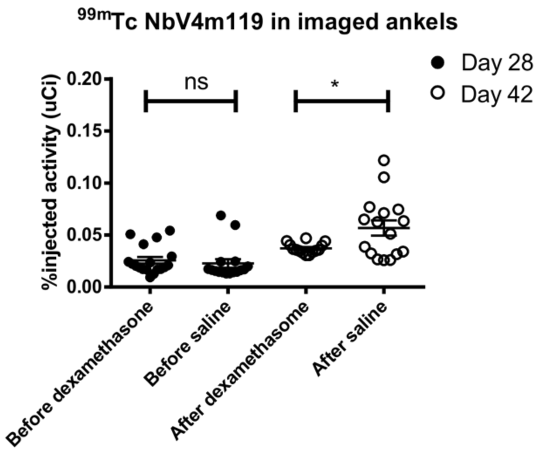

(b)

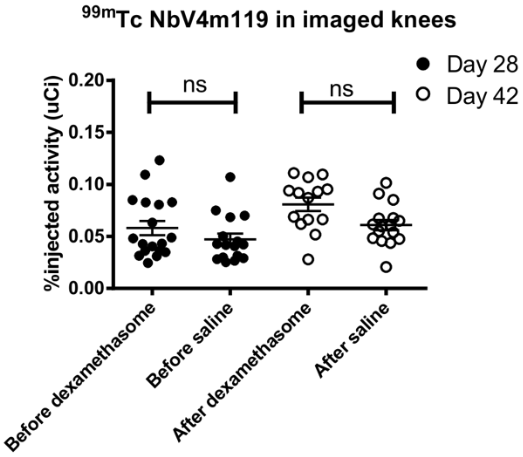

(c)

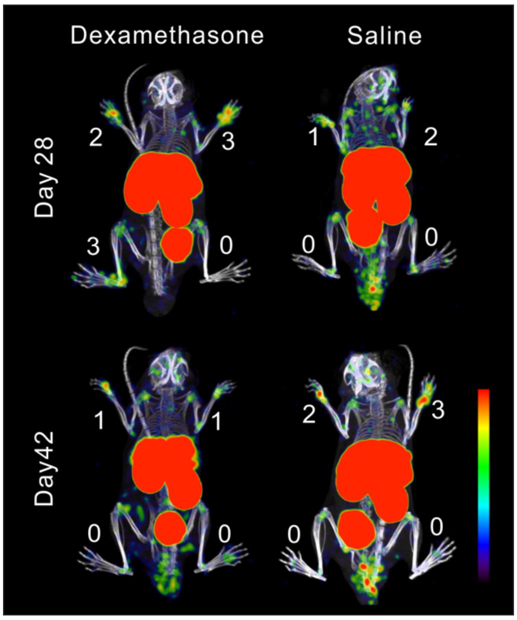

Supplementary Figure 4: (a+b) *SPECT/CT quantification and representative images of ^{99m}Tc-NbV4m119 accumulation in ankles and knees of CIA DBA/1 mice before and after dexamethasone treatment.* Mice were immunized with type II collagen in complete Freund's adjuvant. SPECT/CT imaging was performed on CIA mice 3h post intravenous injection of ^{99m}Tc-V4m119 at 28 (before) and 42 (after) days after immunization. Radioactive signals in regions of interest were quantified for ankles **(a)** and knees **(b)** as % of injected activity (%IA). n=9 arthritic mice per group, mean ± SEM, not significant (ns), *p<0.05, **p<0.01, and ***p<0.001. **(c)** *Representative SPECT/μCT images of ^{99m}Tc-NbV4m119 accumulation in CIA DBA/1 mice before and after dexamethasone treatment*. Clinical scores are indicated next to each joint.
